# Excess breast cancer risk and the role of parity, age at first childbirth and exposure to radiation in infancy

**DOI:** 10.1054/bjoc.2001.1868

**Published:** 2001-08

**Authors:** E Holmberg, L-E Holm, M Lundell, A Mattsson, A Wallgren, P Karlsson

**Affiliations:** The Oncological Centre, Sahlgrenska University Hospital, S-413 45 Göteborg, Sweden; Swedish Radiation Protection Institute, S-171 16, Solna, Sweden; Department of Hospital Physics, Karolinska University Hospital, S-171 76, Stockholm, Sweden; Institution of Oncology-Pathology, Karolinska Institute, Karolinska University Hospital, S-171 76 Radiumhemmet, Stockholm, Sweden; Department of Oncology, Sahlgrenska University Hospital, S-413 45 Göteborg, Sweden

**Keywords:** excess risk, breast neoplasma, ionizing radiation, cohort studies, fertility pattern, haemangioma

## Abstract

Exposure to ionizing radiation is a known risk factor for breast cancer and the fertility pattern is a recognized modifier of breast cancer risk. The aim of this study was to elucidate the interaction between these 2 factors. This study is based on a Swedish cohort of 17 202 women who had been irradiated for skin haemangiomas in infancy between 1920 and 1965. The mean age at treatment was 6 months and the median breast dose was 0.05 Gy (range 0–35.8 Gy). Follow-up information on vital status, parity, age at first childbirth and breast cancer incidence was retrieved through record linkage with national population registers for the period 1958–1995. Analyses of excess relative risk (ERR) models were performed using Poisson regression methods. In this cohort, the fertility pattern differed from that in the Swedish population, with significantly fewer childbirths overall and before 25 years of age but more childbirth after that age. There were 307 breast cancers in the cohort and the standardized incidence ratio (SIR) was 1.22 (95% CI 1.09–1.36). A linear dose–response model with stratification for fertility pattern and menopausal status resulted in the best fit of the data. ERR/Gy was 0.33 (95% CI 0.17–0.53). In absolute terms this means an excess of 2.1 and 5.4 cases per Gy per 10^4^breast-years in the age groups 40–49 and 50–59 years respectively. The fertility pattern influenced the breast cancer risk in this irradiated population in a similar way to that observed in other studies. SIR at dose = 0 was highest, 2.31, among postmenopausal nulliparous women (95% CI 1.48–3.40, *n* = 62). SIR at dose = 0 was lowest in pre- or postmenopausal women with a first childbirth before 25 years of age; 0.89 (0.71–1.09) and 0.88 (0.58–1.25) respectively. Thus, in addition to the dose–effect response in the cohort, part of the breast cancer excess could be explained by a different fertility pattern. The estimates of ERR/Gy for the various categories of age at first childbirth, number of children, menopausal status and ovarian dose were very similar, contradicting any interaction effects on the scale of relative risk. © 2001 Cancer Research Campaign http://www.bjcancer.com


*British Journal of Cancer* (2001) **85**(3), 362–366
© 2001 Cancer Research Campaign
doi: 10.1054/ bjoc.2001.1868, available online at http://www.idealibrary.com on IDEAL
http://www.bjcancer.com

### Excess breast cancer risk and the role of parity, age at first childbirth and exposure to radiation in infancy

**E Holmberg[1], L-E Holm[2], M Lundell[3], A Mattsson[4], A Wallgren[5] and P Karlsson[5]**

[1]The Oncological Centre, Sahlgrenska University Hospital, S-413 45 Göteborg, Sweden; [2]Swedish Radiation Protection Institute, S-171 16, Solna Sweden; [3]Department of Hospital Physics, Karolinska University Hospital, S-171 76 Stockholm, Sweden; [4]Institution of Oncology-Pathology, Karolinska Institute, Radiumhemmet, Karolinska University Hospital, S-171 76 Stockholm, Sweden; [5]Department of Oncology, Sahlgrenska University Hospital, S-413 45 Göteborg, Sweden

**Summary** Exposure to ionizing radiation is a known risk factor for breast cancer and the fertility pattern is a recognized modifier of breast cancer risk. The aim of this study was to elucidate the interaction between these 2 factors. This study is based on a Swedish cohort of 17 202 women who had been irradiated for skin haemangiomas in infancy between 1920 and 1965. The mean age at treatment was 6 months and the median breast dose was 0.05 Gy (range 0–35.8 Gy). Follow-up information on vital status, parity, age at first childbirth and breast cancer incidence was retrieved through record linkage with national population registers for the period 1958–1995. Analyses of excess relative risk (ERR) models were performed using Poisson regression methods. In this cohort, the fertility pattern differed from that in the Swedish population, with significantly fewer childbirths overall and before 25 years of age but more childbirth after that age. There were 307 breast cancers in the cohort and the standardized incidence ratio (SIR) was 1.22 (95% CI 1.09–1.36). A linear dose–response model with stratification for fertility pattern and menopausal status resulted in the best fit of the data. ERR/Gy was 0.33 (95% CI 0.17–0.53). In absolute terms this means an excess of 2.1 and 5.4 cases per Gy per $10^4$ breast-years in the age groups 40–49 and 50–59 years respectively. The fertility pattern influenced the breast cancer risk in this irradiated population in a similar way to that observed in other studies. SIR at dose = 0 was highest, 2.31, among postmenopausal nulliparous women (95% CI 1.48–3.40, $n$ = 62). SIR at dose = 0 was lowest in pre- or postmenopausal women with a first childbirth before 25 years of age; 0.89 (0.71–1.09) and 0.88 (0.58–1.25) respectively. Thus, in addition to the dose–effect response in the cohort, part of the breast cancer excess could be explained by a different fertility pattern. The estimates of ERR/Gy for the various categories of age at first childbirth, number of children, menopausal status and ovarian dose were very similar, contradicting any interaction effects on the scale of relative risk. © 2001 Cancer Research Campaign http://www.bjcancer.com

**Keywords**: excess risk; breast neoplasma; ionizing radiation; cohort studies; fertility pattern; haemangioma

Exposure of the mammary gland to ionizing radiation is one of the best-defined risk factors for breast cancer in women. Irradiation of the breast at younger ages appears to carry a higher relative risk for breast cancer than exposure later in life, and the excess relative risk per Gy (ERR/Gy) seems to decrease with attained age (Baral et al, 1977; Boice et al, 1981, 1991; Shore et al, 1986; Davis et al, 1989; Hoffman et al, 1989; Hrubec et al, 1989; Modan et al, 1989; Tokunaga et al, 1991, 1994). Except for these observations, little is known about ionizing radiation and interactions with other known risk factors for breast cancer. Most of the risk factors for breast cancer are related to endocrine status or events, such as age at menarche, age at first birth, number of pregnancies and age at the menopause. In particular, an early age at the first childbirth protects against breast cancer (MacMahon et al, 1970). It has been suggested that among Japanese women exposed to ionizing radiation, a first full-term pregnancy at an early age may be protective against radiation-related risk (Land et al, 1994). However, reproductive factors and hormone use appear to act independently of radiation exposure on the risk of breast cancer among the same population (Goodman et al, 1997).

*Received 8 December 2000*
*Revised 4 April 2001*
*Accepted 4 April 2001*

*Correspondence to:* E Holmberg

We have previously presented 2 Swedish cohorts who were treated with ionizing radiation in infancy because of haemangiomas and an excess of breast cancer cases was observed, with a dose-dependent ERR of 0.35 per Gy (Lundell et al, 1999). The ERR/Gy was substantially lower than that found in several other studies. In a separate investigation on reproduction outcome in this cohort, it was found that the treated women deviated from the general population by having longer education and by smoking less (Kallen et al, 1998). There were also differences in the number of deliveries in this cohort compared to numbers derived from Swedish rates and there were fewer infants with a birth weight less than 2500 g (Kallen et al, 1998). These factors could be interpreted as an effect of social selection, which might explain part of the excess of breast cancer cases found. It is important to have control of confounding factors like these, especially when the risk related to radiation seems to be low.

The aim of this study was to explore whether the protective effect of early pregnancy and the number of children interact with the effects of previous exposure to ionizing radiation of the infant breast.

### MATERIALS AND METHODS

The cohorts and the treatment have been described in detail elsewhere (Lundell et al, 1999). Children were treated similarly in Stockholm and Gothenburg for skin haemangiomas. In

Stockholm, 9849 female children were irradiated between 1920 and 1959. Of these, 9675 children (98%) who had been treated before the age of 18 months were known to be alive in 1958 and they were followed up. In Gothenburg 7632 female children were treated between 1930 and 1965. From this cohort, 7527 females (98%) fulfilling the same criteria as the Stockholm cohort were followed up.

The haemangiomas were located anywhere on the body surface, but 42% of them were located in the head and neck region and 25% in the thoracic region. The mean age at treatment was 6 months. The children received one to several treatments, with a mean number of 1.5.

### Dosimetry

The dosimetry has been described in detail elsewhere (Lundell, 1994). Briefly, 89% of the cases were treated with radium-226 and in 10% external X-rays were used. Isodose curves and dose-rate tables were used for flat radium applicators and X-ray treatments. When radium needles were used, the dose rate was measured with thermoluminescence dosimeters (TLD) in a phantom corresponding to the size of a 6-month-old child to determine the dose rate to different organs (Lundell, 1994). Adjustment was made for the age of the children (Lundell, 1994). The mean breast dose was 0.29 Gy and the median dose was 0.05 Gy (range 0–35.8 Gy).

### Record linkages

The cohorts were matched by record linkage with 5 national population registers, taking advantage of the unique identification number which is given to all Swedish residents. The registers were:

- The National Population Register, which was used to trace individuals alive and living in Sweden at the end of the study period, i.e. Dec 1995.
- The Emigration Register, established in 1968, which was used to obtain the dates of emigration up until 1995. Information about emigrations before 1968 was obtained from local parishes.
- The Swedish Cause of Death Register, which was established in 1952, which was used to obtain dates of death and underlying causes of death. Information on deaths occurring before 1952 was traced through the local parishes.
- The Swedish Cancer Register (SCR), which was used to obtain information about the occurrence and dates of breast cancer diagnoses in the cohorts between 1958 and 1995. Since the register started in 1958, tumours occurring before that time were not included in the analysis.
- The Fertility Register containing information on deliveries from 1941 and forward was used to obtain information about number and year of deliveries until 1995.

### Statistical methods

Since the absorbed dose differed by breast, the risk calculations were based on breast-years (BY). Bilateral breast cancer was considered as 2 separate events, as in the previous study (Lundell et al, 1999). Breast-years and tumours before 1958, the year when the Swedish Cancer Register was established, were not included in the analysis. To calculate standardized incidence ratios (SIR), expected rates were obtained from the female population of the Country of Stockholm and the West of Sweden Health Care Region, respectively, as most patients were residents of these 2 regions. According to the population census for 1970 and 1990, 53% and 47% of the women in the Stockholm sub-cohort lived in the Country of Stockholm. For the Gothenburg sub-cohort, the proportions were 77% and 73%, respectively. The expected number of cases was calculated by multiplying attained age-, calendar period-and cohort-specific breast-years by the corresponding side-specific breast cancer incidence rates and then summed. The reference incidence rates were stratified by attained age (0–9, 10–19, 20–24, ..., 70–74 and > 75 years of age), calendar periods (1958–1963, 1964–1969, 1970–1975, 1976–1981, 1982–1987 and 1988–1995) and region. Side-specific reference rates were estimated for the period 1958–1969 as this had not been recorded in the SCR. The estimation was done assuming that the age-and side-specific relative distributions between left and right breast during 1958–1969 were identical to the corresponding distributions for the period 1970–1995. The SIR was defined as observed over expected number of cases.

Expected numbers of births and reproduction rates in Sweden for the years 1961–1985 were calculated using tables from the Statistical Year-book for Sweden.

To study dose–response and possible interactions with other factors, Poisson regression models were fitted using the AMFIT program of the Epicure software (Preston et al, 1988–1993). For this analysis, the follow-up time of the individuals was grouped according to cohort into 2 groups, fertility pattern into 5 groups (before any pregnancy, age at first pregnancy <20 years of age, 20–24 years, 25–29 years and ≥30 years), breast dose into 5 categories (<1 cGy, 1–9, 10–99, 100–999 and ≥1000 cGy), attained age into 12 categories (<20 years, 5-year intervals until age 70, ≥70 years of age), and the calendar time into 5-year categories beginning at 1 January, 1958. To the various cells thus created, mean values were assigned (breast dose, ovarian dose, number of children, age at first childbirth, attained age).

In the modelling, the 5 fertility categories were also pooled into 3 categories (age at first childbirth <25 years, age at first childbirth ≥25 years or no childbirth). The menopausal status, pre- or post-menopause, was not known but the age of 50 years was used to define the menopause.

Inferences about dose–response relationships were based on the multiplicative excess relative risk (ERR) models. The observed number of cases over the cells of the table to which the models were fitted were assumed to follow a Poisson distribution with mean F, where $F=E(a, cp, r)*f_0 (g,m) (1+f_1 (bd)\exp[f_2(...)])$ and E is the expected number of cases based on external stratified incidence rates according to age (a), calendar period (cp) and region (r). $f_0$ models SIR unrelated to exposure for fertility pattern-related groups (g) and menopausal status (m). As the previous study did not show any differences between the sub-cohorts, this analysis was performed on aggregated data from both sub-cohorts (Lundell et al, 1999). $f_1$ models the excess relative risk for the breast dose (bd), and the $f_2$ function models the effect of various possible dose modification factors. SIR(bd) = F/E.

In previous studies, we found SIR(bd) = 1.08(1 + 0.35bd) (Lundell et al, 1999). As a check of the validity of the obtained estimates of $f_1$, the Poisson regression analyses were also repeated using internal reference rates with stratification for attained age and calendar period.

*© 2001 Cancer Research Campaign*

Parameter estimates, confidence intervals and tests were computed by maximum likelihood methods.

### RESULTS

The mean age at the end of follow-up was 46 years (range 30–75 years) and there were 1 263 304 BY at risk during the period 1958–1995. A total of 307 invasive breast cancers were diagnosed in 291 women (SIR = 1.22; 95% CI 1.09–1.36).

A total of 206 breast cancers developed before 50 years of age (SIR = 1.08; 95% CI 0.94–1.23) and 101 cases developed at ages above 50 years (SIR = 1.67; 95% CI 1.37–2.02, Figure 1). Although only 4% of the BY in the analysis were after 50 years of age, 33% of the cases occurred in this age group.

### ERR and fertility pattern

The total number of childbirths in the cohort during the period 1961–1995 was 30 513, compared to an expected number of 31 765 (O/E = 0.96, 95% CI 0.95–0.97). Before 25 years of age 8994 childbirths occurred, whereas 12 020 were expected (O/E = 0.75, 95% CI 0.73–0.76). For ages ≥25 years 21 519 childbirths were observed, which was more than expected, 19 744 (O/E = 1.09, 95% CI 1.08–1.11). Table 1 shows a further subdivision according to age at childbirth.

Among nulliparous women, 62 breast cancers occurred (SIR = 1.57; 95% CI 1.21–1.99). In those with a first childbirth before the age of 25 SIR was 0.97 (95% CI 0.81–1.16, $n$ = 116) and in the women with a first childbirth after 25 years of age the SIR was 1.37 (95% CI 1.15–1.63, $n$ = 129). A further subdivision according to age at first childbirth is given in Table 2. The subdivision of fertility pattern into 5 categories in the backgroundterm (f0) did not improve the fit of the ERR-model significantly compared to using 3 categories. The incidence rates of breast cancer in women without childbirths, or according to whether the first childbirth occurred before or after 25 years of age, are shown in Figure 2.

Age at first childbirth as a continuous variable with no children as a separate variable was a significant predictor of the SIR. In this model, the SIR for a first childbirth at the age of 20 was 0.99 (95% CI 0.81–1.19), and it increased by 0.04 (95% CI 0.01–0.07) for each additional year of age at the first childbirth.

**Table 1** Number of childbirths during 1961–1995 among the women in the Swedish haemangioma cohort compared to the Swedish population

| Age at childbirth (years) | Number of children | Expected number of children | Ratio (95% CI) observed/expected |
|---|---|---|---|
| <20 | 1492 | 2616 | 0.57 (0.54–0.60) |
| 20–24 | 7502 | 9405 | 0.80 (0.78–0.82) |
| 25–29 | 10 634 | 10 412 | 1.02 (1.00–1.04) |
| ≥30 | 10 885 | 9332 | 1.17 (1.14–1.19) |
| All ages | 30 513 | 31 765 | 0.96 (0.95–0.97) |

Number of children as a numerical variable also showed a significant correlation to the SIR ($P$ = 0.0061). The SIR for nulliparous women was 1.65 (95% CI 1.31–1.94) and decreased by 0.24 (95% CI 0.07–0.41) for each additional child. The addition of the age at first childbirth to this latter model or the number of childbirths to a model containing age at first childbirth did not significantly improve the fit.

There was a significant interaction effect, $P < 0.001$, in the background term between fertility pattern and menopausal status. Among nulliparous women or in women with a first childbirth after 25 years of age, SIR was almost doubled postmenopausal compared to premenopausal (Table 3).

### ERR and dose–response modelling

The mean breast dose in the cohort was 0.29 Gy (range 0–35.8 Gy). There was no difference in the mean breast dose between nulliparous women (0.28 Gy), women with first childbirths before the age of 25 years (0.29 Gy) and women having their first childbirth after 25 years of age (0.29 Gy). Furthermore, the mean ovarian dose was the same (0.06 Gy) in these 3 groups. The mean dose in the affected breasts was 1.1 Gy (range 0–35.8 Gy).

The simple linear dose–response model was SIR (bd) = 1.10 (1 + 0.33 bd). The intercept value, 1.10 (95% CI 0.97–1.24),

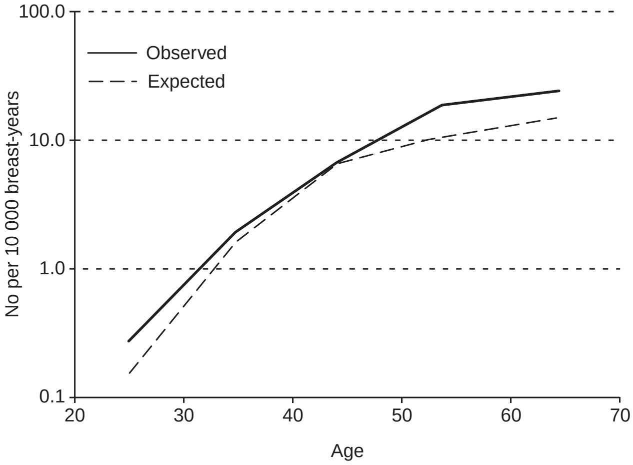

**Figure 1** Observed incidence rate of breast cancer in the cohort and expected incidence rate calculated from age, period and regionally matched data from the Swedish Cancer Register. The standardized incidence ratio was 1.08 before, and 1.67 after age 50

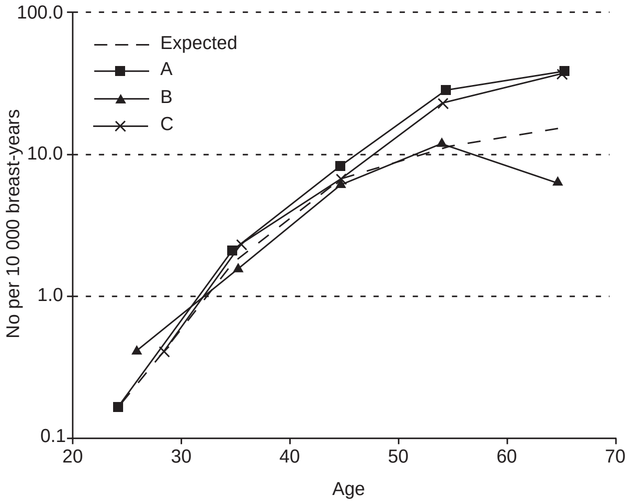

**Figure 2** Observed incidence rate of breast cancer in nulliparous women (A), in women with a first childbirth before the age of 25 (B), and in those with a first childbirth at 25 years of age or thereafter (C). For comparison, the expected incidence rate of breast cancer calculated from age, period and regionally matched data in the Swedish Cancer Register is given

© 2001 Cancer Research Campaign

**Table 2** Observed and fitted statistics by fertility pattern. The multiplicative ERR-model SIR (g) = $f_0$(g) was used to calculate the SIR in the different fertility categories (g)

| Age at first childbirth | Breast cancer cases | Breast-years | SIR (95% CI) |
|---|---|---|---|
| Age at first childbirth <20 | 33 | 115 753 | 1.07 (0.75–1.48) |
| Age at first childbirth, 20–24 | 83 | 277 559 | 0.94 (0.76–1.16) |
| Age at first childbirth, 25–29 | 88 | 175 887 | 1.34 (1.08–1.64) |
| Age at first childbirth ≥30 | 41 | 56 931 | 1.48 (1.07–1.98) |
| Nulliparous | 62 | 637 172 | 1.57 (1.21–2.00) |
| Total | 307 | 1 263 304 | 1.22 (1.09–1.36) |

**Table 3** Fitted statistics by age at fertility pattern (g), menopausal status (m) and breast dose (bd). The multiplicative ERR-model SIR (g, m, bd) = $f_0$ (g,m) (1+$f_1$ (bd)) was used. The first SIR column is for a model without the dose term ($f_1$(bd)). In the second SIR column the model has the dose term included and ERR/Gy (95% CI) was 0.33 (0.17–0.53)

| Fertility pattern | Menopausal status | SIR (g,a) (95% CI) | SIR (g,a,bd = 0) (95% CI) |
|---|---|---|---|
| Age at first childbirth <25 | Pre- | 0.98 (0.79–1.20) | 0.98 (0.71–1.09) |
| Age at first childbirth <25 | Post- | 0.97 (0.65–1.38) | 0.88 (0.58–1.25) |
| Age at first childbirth ≥25 | Pre- | 1.12 (0.89–1.38) | 1.01 (0.80–1.26) |
| Age at first childbirth ≥25 | Post- | 2.17 (1.62–2.81) | 1.93 (1.44–2.53) |
| Nulliparous | Pre- | 1.28 (0.92–1.73) | 1.17 (0.83–1.58) |
| Nulliparous | Post- | 2.56 (1.65–3.75) | 2.31 (1.48–3.40) |

described the SIR in the cohort not related to breast dose (SIR (bd = 0)). The coefficient of bd, 0.33 (95% CI 0.18–0.54), is the ERR/Gy.

Taking the fertility pattern and menopausal status into account in the background gave the same ERR/Gy, 0.33 (95% CI 0.17–0.53). SIR (bd = 0) varied from 0.88 to 2.31 in the different background categories (Table 3). Inclusion of these background categories in the model significantly improved the fit of the data ($P$ = 0.02).

Estimates of excess cases per unit dose and $10^4$ breast-years (EAR) were derived from the fitted ERR-model. There were 2.1 (95% CI 1.2–3.2) and 5.4 (95% CI 3.0–8.3) excess cases per Gy per $10^4$ breast-years in the attained age groups 40–49 and 50–59 years respectively.

**Table 4** Modification factors for breast dose response in the multiplicative ERR-model, SIR (g,m,bd,...) = $f_0$(g,m)(1+$f_1$(bd)exp($f_2$(...)). The term $f_0$(g,m) models the modification of background due to the fertility pattern (g) and menopausal status (m). The term $f_1$(bd) models the ERR/Gy and $f_2$(...) the modification factors of the dose response

| Variables in $f_2$(...) | ERR/Gy (95% CI) | *P* value |
|---|---|---|
| With no modification factor | 0.33 (0.17–0.53) | |
| Fertility pattern, 3 categories | | 0.85 |
| Age at first childbirth <25 | 0.29 (0.08–0.64) | |
| Age at first childbirth ≥25 | 0.31 (0.10–0.65) | |
| Nulliparous | 0.44 (0.10–1.07) | |
| Number of children, 2 categories | | 0.58 |
| Nulliparous | 0.44 (0.10–1.07) | |
| One or more child | 0.30 (0.14–0.53) | |
| Menopausal status, 2 categories | | 0.16 |
| Pre- | 0.43 (0.21–0.75) | |
| Post- | 0.18 (-0.04–0.47) | |
| Ovarian dose, continuous | | 0.63 |
| Null dose | 0.31 (0.15–0.56) | |
| Change per cGy | 0.7% (-3.1%–3.0%) | |

The percentage of cases among those who received doses in excess of 0.01 Gy ($AR_{0.01GY}$) that can be attributed to radiation was 12.4% (95% CI 6.9–18.7%).

Poisson regression analyses without external reference rates but with adjustment for attained age and calendar period gave identical estimates of ERR/Gy in the total group and in all analysed subgroups

### Interaction effects of dose response

Table 4 gives the results of the influence on the ERR/Gy of several factors: fertility pattern, number of children, menopausal status and ovarian dose. None of these significantly modified the dose response nor did age at first childbirth or attained age as continuous variables.

### DISCUSSION

Irradiation of the female breast in infancy increases the risk for breast cancer later in life (Lundell et al, 1999). In this study there was a 22% excess of breast cancer cases compared with expected values using data from the counties where most of the individuals lived. This group of exposed girls was not a representative sample of the underlying population with respect to age at childbirth, since they had fewer childbirths overall and especially fewer childbirths before the age of 25 as measured in 1961–1995 (Table 1). Part of the recorded excess of breast cancer might be attributed to this since one of the strongest risk factors for breast cancer is a late age at the first pregnancy (MacMahon et al, 1970). In our study, a first pregnancy before the age of 25 years conferred protection against breast cancer, which became most obvious after the age of 50 (Figure 2 and Table 3).

It has been suggested that a childbirth at an early age could not only protect against breast cancer but more specifically protect against the radiation-related risk (Land et al, 1994). Although we found no significant interaction between fertility pattern and the ERR/Gy, the low power of the study does not exclude an interaction. ERR/Gy was, however, similar in the categories of women

*© 2001 Cancer Research Campaign* 

divided according to fertility pattern (Table 4).

Information about the deliveries was collected from the Swedish Fertility Register, which was set up during the early 1960s and contained information about all children who lived at home at that time. All later childbirths are included in the register. This means that in some of the oldest individuals in our study, an early pregnancy may have been missed. Such individuals might therefore have been included among women without pregnancies or with a first childbirth after the age of 25. This would reduce the differences in breast cancer rates between the 3 groups defined by the presence of a pregnancy or by age at first childbirth. It would possibly also reduce the power to detect interactions between the effect of radiation and the protective effect of the age at first childbirth on the breast cancer risk. Taken together, these factors would tend to diminish any differences between the groups.

The ERR/Gy tended to be lower after the age of 50 years (postmenopausal) than before (premenopausal), although this difference was not statistically significant ($P$ = 0.16). In many other studies, there has also been a tendency for the relative risk to diminish after 45–50 years of age (BEIR-V, 1990), which might indicate that the risk for radiation-induced cancers diminish with time since exposure. In our previous study (Lundell et al, 1999) where we did not adjust for the fertility pattern and menopausal status, we found no indication of flattening of the risk curve for radiation-related breast cancer. However considering that the overall increased SIR after age 50 may reflect the fertility pattern in this cohort, it is possible that the effect of ionizing radiation on the breast cancer risk decreases after the age of 50, as has been suggested in other studies.

In the cohort of A-bomb survivors the ERR/Sv was 4.6 among those who were 0–4 years old at time of the bombings (Tokunaga et al, 1994). The A-bomb survivors showed a decreasing ERR/SV by increasing age at exposure. Thus, for the entire group of A-bomb survivors who were 0–19 years at the time of exposure, ERR/Sv was 2.7 and in the order of 10 times higher than that of our cohort. It has been suggested that the protracted low dose rate treatment as in our cohort might ameliorate the dose effect (Howe and McLaughlin, 1996; Lundell et al, 1999). Although a relative risk model for dose-responses in breast cancer is generally preferred (BEIR-V, 1990), it does not entirely account for differences between cohorts with very different background incidence levels. On the absolute scale the excess number of cases per Gy per $10^4$ person-years were only double in the A-bomb survivors aged 0–19 years at the time of exposure (17.2) to that of our cohort (7.8). Therefore, it is important to specify the model when comparing estimates between populations with different baseline rates. In conclusion, our material was probably selected for a fertility pattern with fewer childbirths at early ages. This may explain part of the observed excess of breast cancers, especially after the age of 50, in this cohort of children that were exposed to ionizing irradiation in infancy. However, the estimates of ERR/Gy for the different categories of age at first childbirth were rather similar and the fertility pattern did not show any significant interaction on the excess risk of cancer after irradiation. Obviously, the power to detect an interaction was low, especially at attained age over 50 years. Since only 4% of the breast-years were after this age, further follow-up will rapidly improve the power.

### ACKNOWLEDGEMENTS

This study was supported by grants from the Swedish Radiation Protection Institute (P886.95) and the European Commission under contract F14P-CT95-0009.

### REFERENCES

Baral E, Larsson LE and Mattsson B (1977) Breast cancer following irradiation of the breast. *Cancer* **40**: 2905–2910

Beir V (1990) *Health effects of exposure to low levels of ionizing radiation* National Research Council, National Academy Press

Boice JD, Monson RR and Rosenstein M (1981) Cancer mortality in women after repeated fluoroscopic examinations of the chest. *J Natl Cancer Inst* **66**: 863–867

Boice JD, Jr, Preston D, Davis FG and Monson RR (1991) Frequent chest X-ray fluoroscopy and breast cancer incidence among tuberculosis patients in Massachusetts. *Radiat Res* **125**: 214–222

Davis FG, Boice JD, Jr, Hrubec Z and Monson RR (1989) Cancer mortality in a radiation-exposed cohort of Massachusetts tuberculosis patients. *Cancer Res* **49**: 6130–6136

Goodman MT, Cologne JB, Moriwaki H, Vaeth M and Mabuchi K (1997) Risk factors for primary breast cancer in Japan: 8-year follow-up of atomic bomb survivors. *Prev Med* **26**: 144–153

Hoffman DA, Lonstein JE, Morin MM, Visscher W, Harris B.S.d. and Boice JD, Jr. (1989) Breast cancer in women with scoliosis exposed to multiple diagnostic x rays. *J Natl Cancer Inst* **81**: 1307–1312

Howe GR and McLaughlin J (1996) Breast cancer mortality between 1950 and 1987 after exposure to fractionated moderate-dose-rate ionizing radiation in the Canadian fluoroscopy cohort study and a comparison with breast cancer mortality in the atomic bomb survivors study. *Radiat Res* **145**: 694–707

Hrubec Z, Boice JD, Jr, Monson RR and Rosenstein M (1989) Breast cancer after multiple chest fluoroscopies: second follow-up of Massachusetts women with tuberculosis. *Cancer Res* **49**: 229–234

Kallen B, Karlsson P, Lundell M, Wallgren A and Holm LE (1998) Outcome of reproduction in women irradiated for skin hemangioma in infancy. *Radiat Res* **149**: 202–208

Land CE, Hayakawa N, Machado SG, Yamada Y, Pike MC, Akiba S and Tokunaga M (1994) A case-control interview study of breast cancer among Japanese A-bomb survivors II Interactions with radiation dose. *Cancer Causes Control* **5**: 167–176

Lundell M (1994) Estimates of absorbed dose in different organs in children treated with radium for skin hemangiomas. *Radiat Res* **140**: 327–333

Lundell M, Mattsson A, Karlsson P, Holmberg E, Gustafsson A and Holm LE (1999) Breast cancer risk after radiotherapy in infancy: a pooled analysis of two Swedish cohorts of 17,202 infants. *Radiat Res* **151**: 626–632

MacMahon B, Cole P, Lin TM, Lowe CR, Mirra AP, Ravnihar B, Salber EJ, Valaoras VG and Yuasa S (1970) Age at first birth and breast cancer risk. *Bull World Health Organ* **43**: 209–221

Modan B, Chetrit A, Alfandary E and Katz L (1989) Increased risk of breast cancer after low-dose irradiation [published erratum appears in Lancet 1989 Apr 22; 1 (8643): 916]. *Lancet* **1**: 629–631

Preston D, Lubin JH, Pierce DA and McConney ME (1988–1993). *EPICURE User's Guide* Hirsoft International Corp: Seattle

Shore RE, Hildreth N, Woodard E, Dvoretsky P, Hempelmann L and Pasternack B (1986) Breast cancer among women given X-ray therapy for acute postpartum mastitis. *J Natl Cancer Inst* **77**: 689–696

Tokunaga M, Land CE and Tokuoka S (1991) Follow-up studies of breast cancer incidence among atomic bomb survivors. *J Radiat Res (Tokyo)* **32 Suppl**: 201–211

Tokunaga M, Land CE, Tokuoka S, Nishimori I, Soda M and Akiba S (1994) Incidence of female breast cancer among atomic bomb survivors, 1950–1985. *Radiat Res* **138**: 209–223

*© 2001 Cancer Research Campaign*
